# A phase II study of anlotinib plus whole brain radiation therapy for patients with NSCLC with multiple brain metastases

**DOI:** 10.1080/07853890.2024.2401618

**Published:** 2024-10-24

**Authors:** Dayong Gu, Hongliang Yu, Naixin Ding, Jianhua Xu, Pudong Qian, Jun Zhu, Ming Jiang, Hua Tao, Xiangzhi Zhu

**Affiliations:** a Department of Radiation Oncology, The Affiliated Cancer Hospital of Nanjing Medical University & Jiangsu Cancer Hospital & Jiangsu Institute of Cancer Research, Nanjing, People’s Republic of China; b Department of Thoracic Surgery, The Affiliated Cancer Hospital of Nanjing Medical University & Jiangsu Cancer Hospital & Jiangsu Institute of Cancer Research, Nanjing, People’s Republic of China

**Keywords:** Whole-brain radiotherapy, anlotinib, non-small cell lung cancer, brain metastasis, intracranial progression-free survival

## Abstract

**Background:**

Whole brain radiotherapy (WBRT) is the mainstay of treatment for patients with non-small cell lung cancer (NSCLC) with multiple brain metastases (BMs); however, the BRAIN study showed that the efficacy of WBRT is unsatisfactory. This prospective phase II study aimed to evaluate the efficacy and safety of WBRT combined with anlotinib, a novel anti-angiogenic multi-target tyrosine kinase inhibitor (TKI), in patients with multiple BMs (>3) from advanced NSCLC.

**Methods:**

Patients with advanced NSCLC with multiple BMs who had received two or more lines of treatment were eligible for enrolment into this study. All patients were treated with anlotinib (8–12 mg, QD, on days 1–14 of a 21-day cycle) combined with WBRT (DT 30 Gy/12 F), followed by maintenance therapy with anlotinib until disease progression or treatment intolerance. The primary endpoint of this study was the intracranial progression-free survival (iPFS). The secondary endpoints were intracranial objective response rate (iORR), intracranial disease control rate (iDCR), overall survival (OS) and treatment safety.

**Results:**

Between May 2019 and January 2021, 28 patients were enrolled, all of whom were evaluable for efficacy and safety. The median age was 57.7 years, and 46.4% were male. Twenty-five patients had adenocarcinoma (89.3%), six had EGFR mutations (21.4%) and two had ALK mutations (7.1%). The median iPFS was 11.1 months (95% confidence interval (CI): 5.4–16.8 months) and the median OS was 13.4 months (95% CI: 5.2–21.6 months). The iORR was 71.4% (six complete responses + 14 partial responses). The most frequently observed adverse events (AEs) were hypertension (71.4%), fatigue (64.3%), anorexia (46.4%), and foot and hand skin reactions (25.0%). No patients developed ≥ grade 4 AEs. No intracranial haemorrhages occurred during treatment. Dose adjustment due to AEs occurred in 17.9% of patients.

**Conclusions:**

Anlotinib combined with WBRT is effective and well-tolerated in patients with NSCLC with multiple BMs.

## Introduction

Brain metastasis (BM) is a common clinical condition that severely affects the prognosis of patients with non-small cell lung cancer (NSCLC). BM presents in approximately 20–65% of patients with NSCLC during the course of disease treatment [1]. Patients with NSCLC who suffer from BMs have a poor prognosis, with an average overall survival (OS) of approximately 2–3 months if left untreated [2]. For the treatment of BMs, stereotactic radiosurgery (SRS) is usually performed if the number of metastases is limited (≤3) and the lesions are <3 cm in diameter. For multiple BMs (>3), although there are studies supporting the use of SRS in multiple BMs scenario, whole brain radiotherapy (WBRT) remains more widely accepted treatment modality [3–5]. However, the results of the BRAIN study showed that the local control rate for WBRT is still low at approximately 24–55%, and the median OS is approximately 5 months [5–8]. Therefore, new effective sensitization regimens to improve the efficacy in such patients deserve further investigation.

Recent studies have shown that anti-angiogenic drugs can improve the oxygenation of tumour cells and enhance their sensitivity to radiotherapy by normalizing intratumoural blood vessels [9,10]. Anlotinib is a novel multitarget antiangiogenic tyrosine kinase inhibitor (TKI) approved for the treatment of NSCLC and had already shown vibrant activity in hindering BMs [11,12]. Therefore, we hypothesized that, as an antiangiogenic agent, anlotinib, could improve the radiosensitivity to WBRT and improve control of multiple BMs. This prospective phase II study was designed to examine the efficacy and safety of anlotinib in combination with WBRT during third-line treatment for the control of multiple BMs (>3) from NSCLC. Our preliminary results have already been reported at the ASCO annual conference in 2021 [13], this manuscript presents the 2-year follow-up results of this study.

## Patients and methods

### Patients

Patients with multiple BMs from NSCLC admitted to our hospital between May 2019 and January 2021 were screened for inclusion in the study. The main eligibility criteria for inclusion were as follows: patients aged ≥18 years; patients who had received first- and second-line standard treatment (surgery/thoracic radiotherapy/chemotherapy) and/or targeted therapy, and formed drug resistance, with anlotinib administered as the third-line treatment; ≥4 intracranial metastatic lesions with at least one that could be measured by enhanced magnetic resonance imaging (MRI) scan; and expected survival of ≥2 months. The main exclusion criteria were as follows: meningeal metastasis and unsatisfactory blood pressure control (systolic blood pressure (>150 mmHg), diastolic blood pressure (>100 mmHg) and serum creatinine >1.2 times the upper limit of normal value). Further details can be found on the trial registration website.

### Study design

This study was a prospective, single-arm, phase II clinical trial that was registered at the Chinese Clinical Trial Registry Center with the trial ID ChiCTR 1900022093. After the patients were enrolled in the study, they were treated with anlotinib (8–12 mg, QD, days 1–14 of a 21-day cycle) combined with WBRT (30 Gy/12 F), followed by maintenance therapy with anlotinib until disease progression or the development of treatment intolerance. In this study, the dose reduction protocol for anlotinib involved the following steps: if a patient experienced adverse reactions related to anti-angiogenic medication, such as hypertension or skin toxicity (as judged by the physician), the dosage could be decreased by one level (10 mg). If intolerance persisted, the dosage could be further reduced to 8 mg. Should the adverse reactions still remain intolerable, the medication would be discontinued.

During the implementation of radiotherapy in the study, the patients were immobilized with a thermoplastic mask and large-aperture computed tomography (CT) was used to scan the cranial brain. The whole brain was set as the clinical target volume (CTV) and the planning target volume (PTV) was created by expanding the CTV by 3 mm in all directions. The prescribed dose was the conventional radiotherapy regimen with the PTV receiving 30 Gy in 12 fractions at five fractions per week. A linear accelerator was used to deliver the radiotherapy.

The primary endpoint of this study was the intracranial progression-free survival (iPFS). The secondary endpoints were intracranial objective response rate (iORR), intracranial disease control rate (iDCR), OS and treatment safety. The iPFS was assessed from the time of WBRT initiation with oral anlotinib administration to the time of detection of intracranial lesion progression. The iORR was defined as the sum of the proportion of patients achieving intracranial tumour CR or PR according to the Response Evaluation Criteria in Solid Tumors (RECIST). The iDCR was defined as the sum of the proportion of patients achieving intracranial tumour CR, PR and SD. OS was defined as the time from WBRT to death from any cause. Tumour response was assessed every 8 weeks using MRI, until disease progression. Intracranial tumour progression was defined as an enhanced MRI showing enlarged lesions or new intracranial metastases. Safety was evaluated based on the toxic effects, which were assessed according to the National Cancer Institute Common Terminology Criteria (version 4.0).

### Statistical analysis

Statistical analysis was performed using SPSS (version 22.0; SPSS Inc., Chicago, IL). Patient survival was estimated using the Kaplan–Meier method. Two-sided tests were used to analyse significant differences. Statistical significance was set at *p* < .05. For the sample size estimation in this prospective phase II clinical trial, historical data showed that the iPFS of patients with multiple BMs after resistance to standard conventional therapy or targeted therapy was approximately 5 months after receiving WBRT alone [8]; and based on the available evidence of the vibrant activity of anlotinib on BM [12], we expected the iPFS after anlotinib combined with WBRT was approximately 9 months. Using the one-sided test, *α* = 0.05, *β* = 0.2 (power = 80%), the sample estimation was performed by using one-sample logrank model via PASS (version 15.0.1; NCSS Inc., Kaysville, UT) software. Results showed that 25 patients were needed. Considering the loss of follow-up and dropout, 28 patients are expected to be enrolled in this study.

## Results

### Patient characteristics

**Figure 1.**
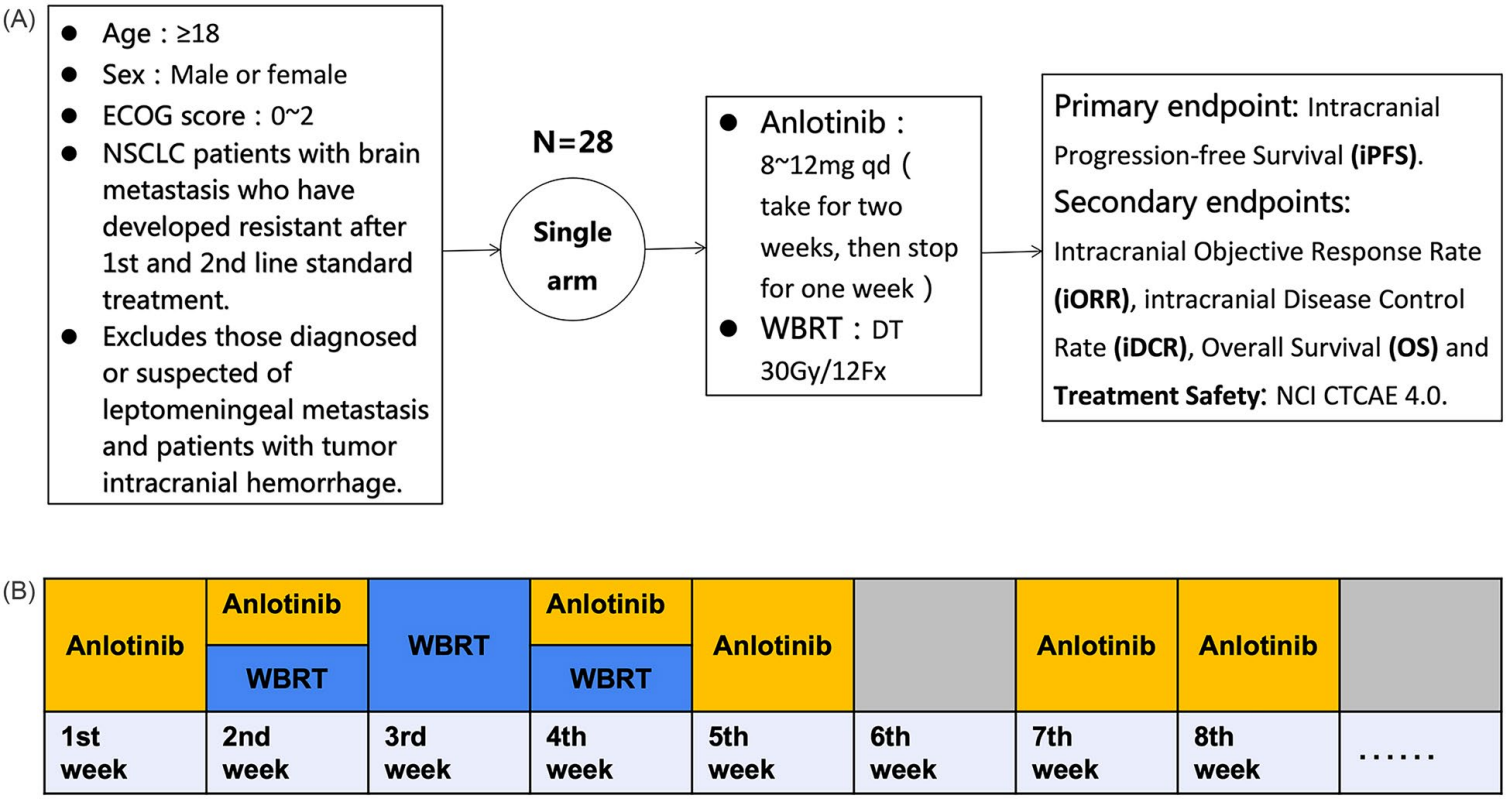
The schematic diagram and trial plan for the use of anlotinib in this clinical trial.

A total of 28 patients were recruited between May 2019 and January 2021. All patients included in this study were newly diagnosed with BMs during follow-up examinations after first-line and second-line systemic treatments, and none had received local treatment prior to inclusion in this study. Of the 28 patients, the median follow-up time was 23.4 (95% confidence interval (CI): 13.9–33.0) months. The median age was 57.7 years, 46.4% were male, 25 patients were diagnosed with adenocarcinoma and three with squamous cell carcinoma. Eight had sensitive EGFR mutations, two had ALK mutations and 18 had no detected mutations. Detailed patient characteristics are shown in Table 1. Detailed schematic diagram and trial plan for the use of anlotinib in this clinical trial are shown in Figure 1(A,B).

**Table 1.** Patient characteristics (*n* = 28).

Characteristics	No. of patients	
Age (years)		
<60	17	60.7%
≥60	11	39.3%
Sex		
Male	13	46.4%
Female	15	53.6%
ECOG PS		
0–1	13	46.4%
2	15	53.6%
Pathology		
Adenocarcinoma	25	89.3%
Squamous cell carcinoma	3	10.7%
Gene mutation		
EGFR	6	21.4%
ALK	2	7.1%
Wild type	12	42.9%
Not detected	8	28.6%
Previous treatment		
TKIs	8	28.6%
ICIs	15	53.6%
Chemotherapy	24	85.7%
Diameter of lesion (cm)		
<2	12	42.9%
≥2	16	57.1%
GPA score		
<2	21	75.0%
≥2	7	25.0%
Anlotinib dose		
12 mg	23	82.1%
10 mg	2	7.1%
8 mg	3	10.7%

### Response of brain metastases

**Figure 2.**
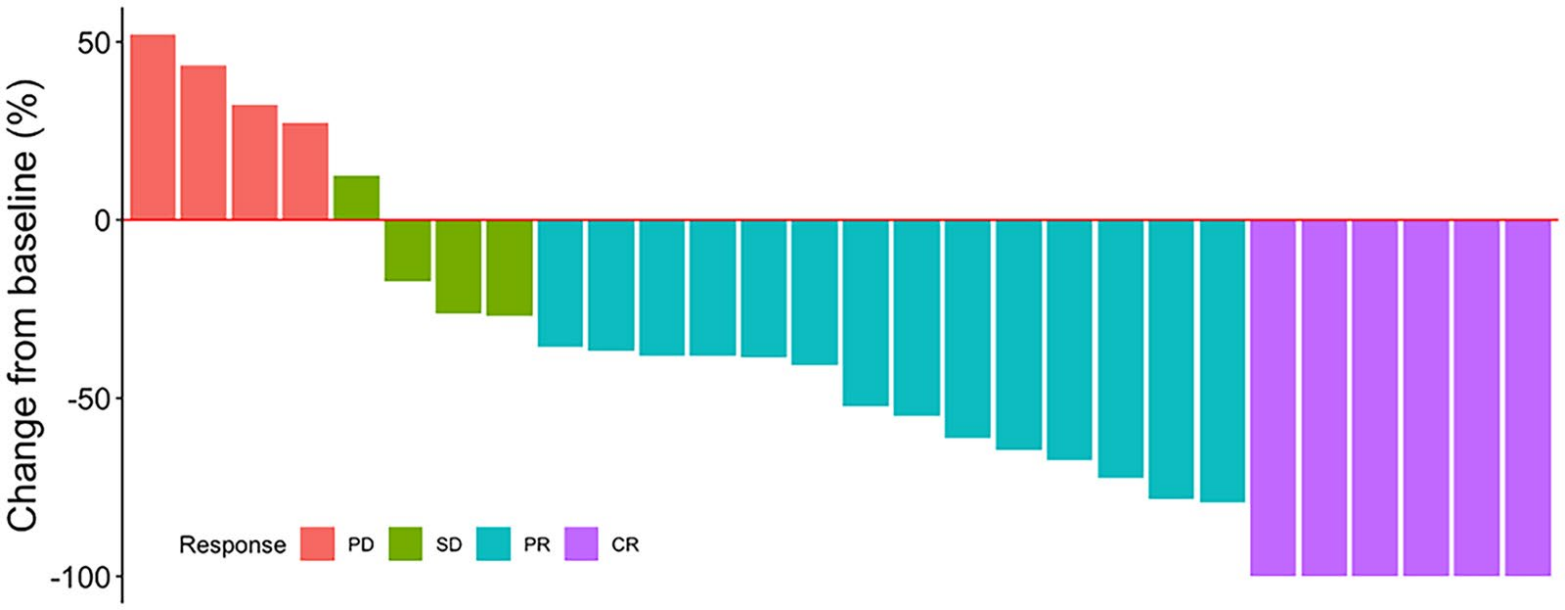
Waterfall chart of intracranial lesion regression after treatment.

**Figure 3.**
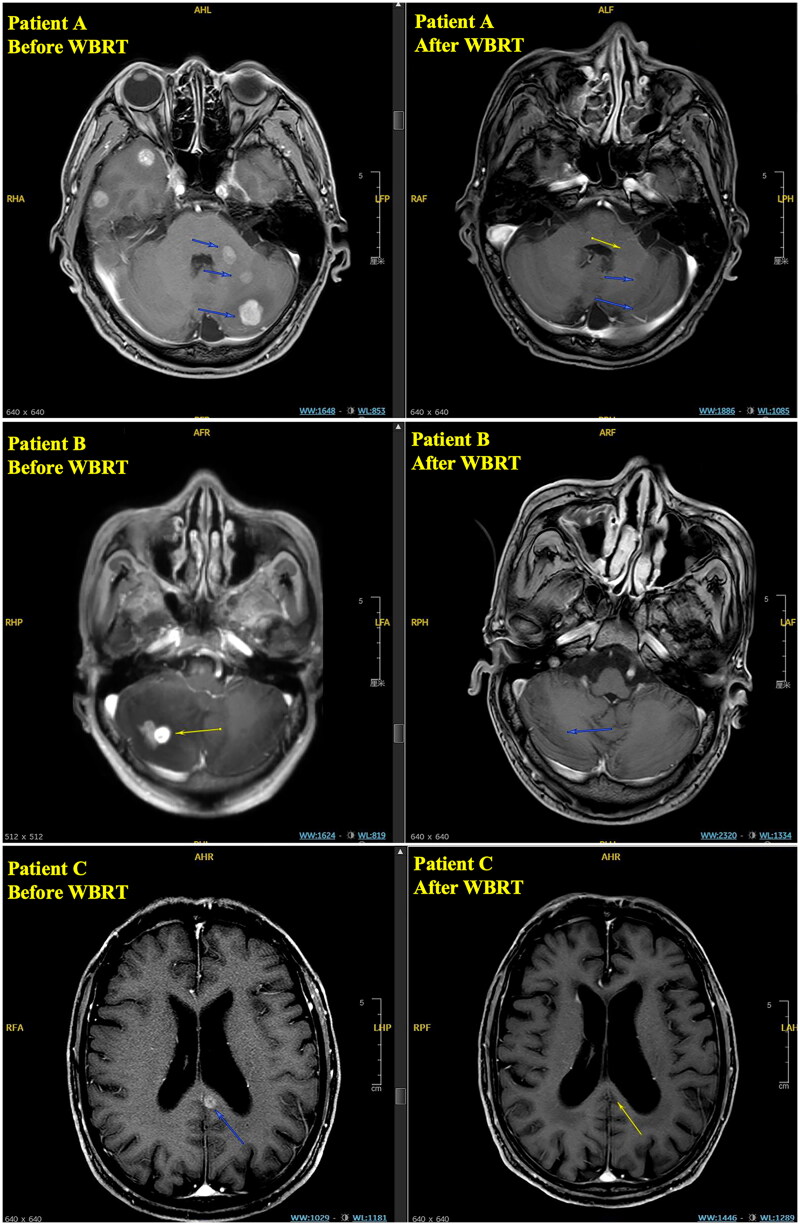
The enhanced T1-weighted MRIs of three representative patients who achieved CR response in intracranial lesions.

A total of 28 patients completed the trial protocol and were followed up, and MRIs were performed to determine the treatment response. The results of the intracranial lesion responses are shown in Table 2. Our data showed that anlotinib combined with WBRT showed very promising control of brain metastatic lesions, with an iORR of 71.4% (six CR + 14 PR), whereas the iDCR of intracranial lesions was 85.7%. A detailed waterfall diagram of tumour regression is shown in Figure 2. The enhanced T1-weighted MRIs showing the tumour size before and after WBRT plus anlotinib treatment from three representative patients who achieved CR are presented in Figure 3.

**Table 2.** Intracranial lesion response.

Response	Cases (%)
CR	6 (21.4%)
PR	14 (50.0%)
SD	4 (14.3%)
PD	4 (14.3%)
iORR	20 (71.4%)
iDCR	24 (85.7%)

The left panels show enhanced T1-weighted MRIs before WBRT, and the right panels show follow-up MRIs of the same brains at the same level slice.

**Figure 4.**
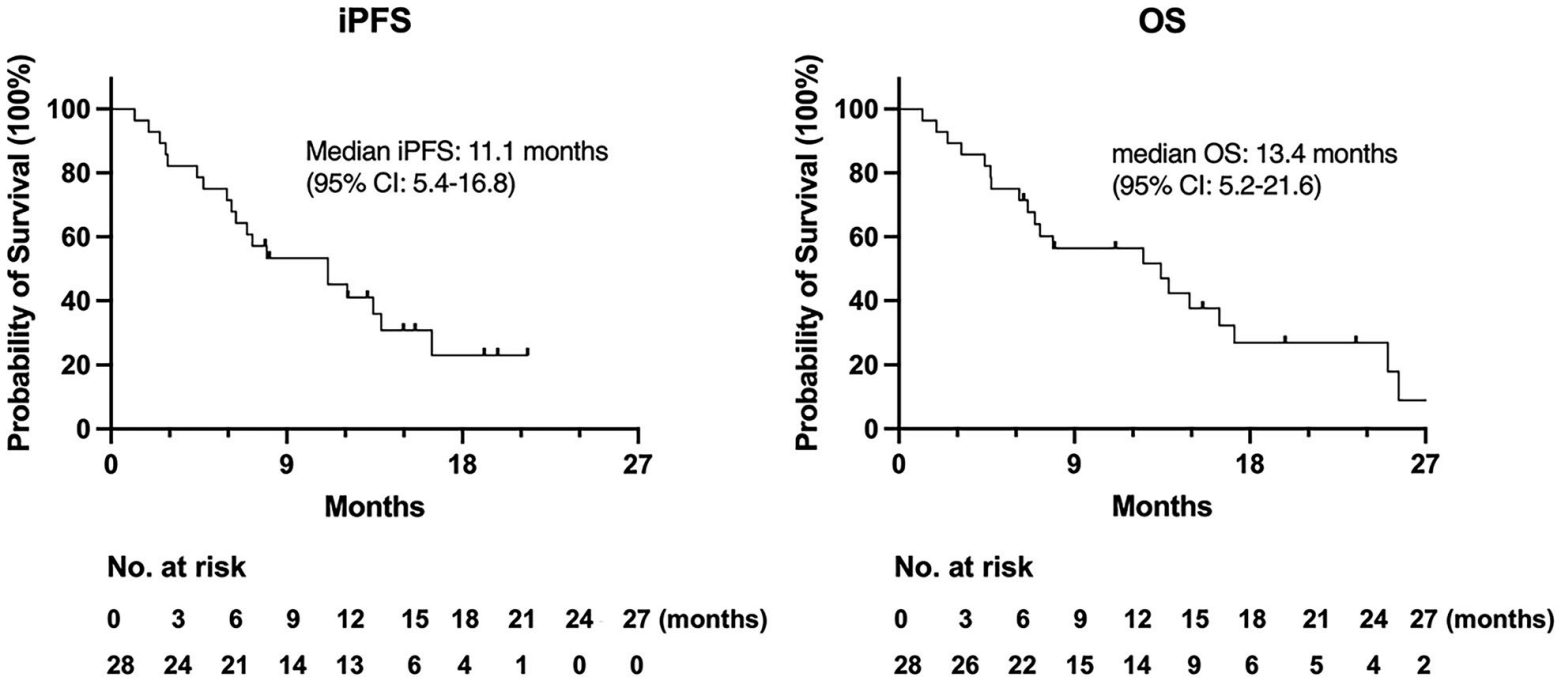
Kaplan–Meier’s survival curves of iPFS and OS.

As of the last follow-up visit in March 2023, 21 patients have died. This study showed that anlotinib combined with WBRT for patients with advanced NSCLC with multiple BMs who had received two or more lines of previous treatments obtained a median iPFS of 11.1 months (95%CI: 5.4–16.8 months) and a median OS of 13.4 months (95% CI: 5.2–21.6 months), respectively. The Kaplan–Meier survival curves are presented in Figure 4. Our preliminary results exceeded our expected median iPFS of 9 months and reached 11.1 months, demonstrating good synergistic effects of anlotinib with WBRT for these NSCLC patients with multiple BMs.

### Adverse events (AEs) and treatment compliance

The AEs were recorded in the present study. None of the patients developed AEs of ≥ grade 4. The most frequently observed AEs were hypertension (71.4%), fatigue (64.3%), anorexia (46.4%) and skin reactions on the feet and hands (25.0%). The most common grade 3 AEs were hypertension (10.7%) and fatigue (7.1%). In addition, we observed one case each of thrombosis and haematuria. No intracranial haemorrhagic events were observed in the present study. Five patients underwent dose-reduction adjustments due to AEs. The detailed recorded AEs are listed in Table 3. In summary, adverse reactions were well tolerated in this study.

**Table 3.** Treatment-related adverse events.

AE	Grade 1–2 (%)	Grade 3 (%)	Total (%)
Hypertension	17 (60.7%)	3 (10.7%)	20 (71.4%)
Fatigue	16 (57.1%)	2 (7.1%)	18 (64.3%)
Anorexia	12 (42.9%)	1 (3.6%)	13 (46.4%)
Foot and hand skin reaction	6 (21.4%)	1 (3.6%)	7 (25.0%)
Vomiting	5 (17.9%)	1 (3.6%)	6 (21.4%)
Headache	4 (14.3%)	0 (0)	4 (14.3%)
Diarrhoea	3 (10.7%)	0 (0)	3 (10.7%)
Leukopenia	1 (3.6%)	0 (0)	1 (3.6%)
Cough	1 (3.6%)	0 (0)	1 (3.6%)
Thrombosis	0 (0)	1 (3.6%)	1 (3.6%)
Haematuria	0 (0)	1 (3.6%)	1 (3.6%)

## Discussion

The prognosis of patients with NSCLC with multiple BMs is poor, and the efficacy of WBRT is unsatisfactory [1, 5, 6]. Studies on WBRT combined with conventional chemotherapy have long failed to improve efficacy because of the blood–brain barrier [14–16]. A series of new treatment modalities used in combination with WBRT have been investigated to improve the outcomes of these patients [17–19]. To date, several lines of evidence have shown that the combination of antiangiogenic agents and radiotherapy has a synergistic sensitizing effect, which is a potential strategy for improving the efficacy of WBRT in these patients [9, 20–22].

Anlotinib is a novel multitarget antiangiogenic TKI. In the phase III clinical trial ALTER0303, anlotinib showed significant antitumour activity against advanced NSCLC [11]. Additionally, subgroup analysis of the trial showed that anlotinib alone had significant activity in controlling intracranial lesions, with a median iORR of 14.3% and a median iDCR of 85.7% in patients with NSCLC with BM [12]. Moreover, anlotinib was associated with significantly longer iPFS compared with placebo, with a hazard ratio of 0.11 (95% CI: 0.03–0.41, *p* = .001) [12]. Thus, we hypothesized that anlotinib may have a synergistic sensitizing effect on WBRT and may improve the therapeutic efficacy in multiple intracranial lesions. However, anlotinib is not always safe and effective under all circumstances. For example, the ALTER-L042 trial showed significant pulmonary toxicity when anlotinib was used concurrently with radiotherapy for lung cancer [23]. Therefore, we conducted this prospective phase II clinical trial to explore the efficacy and safety of anlotinib in combination with WBRT for treating patients with multiple intracranial lesions. Our preliminary results have already been reported at the ASCO annual conference in 2021 [13]. This manuscript presents the 2-year follow-up results of this study.

The results of our study confirmed that anlotinib combined with WBRT had a very promising effect on multiple BMs in NSCLC, with an iORR of 71.4% and an iDCR of intracranial lesions of 85.7%. The median iPFS was 11.1 months (95% CI: 5.4–16.8 months) and the median OS was 13.4 months (95% CI: 5.2–21.6 months). Although this was a single-arm study and cannot be directly compared with the results of other studies, retrospective analysis of previously published studies of WBRT for BMs from NSCLC reported a median iPFS of 2.3–8.8 months [5, 24, 25], which is substantially shorter than that of 11.1 months for anlotinib combined with WBRT in this study.

Similar to this study, another phase II study on WBRT combined with anlotinib for multiple BMs from NSCLC without targetable driver mutations was recently published [26]. In their study, the iORR and iDCR were 66.7% and 90.5%, respectively. The median iPFS and OS were 10.3 and 13.4 months, respectively. The results of the two independent trials mirrored each other and confirmed the effectiveness of anlotinib in combination with WBRT for the treatment of multiple BMs from NSCLC. However, there are some differences between the two trials. First, it should be noted that this study was registered on the clinical trial website prior to that one. Second, the sample size in our study was larger than that in theirs (28 vs. 21) and third, the status of targetable driver mutations was not a prerequisite for enrolling patients in this study. Our preliminary results suggested that EGFR mutations have little effect on the efficacy of anlotinib in combination with WBRT. This is not surprising, as the subgroup analysis of the previous ALTER0303 trial also showed that anlotinib provides survival benefits in NSCLC patients independent of the EGFR mutation status [27].

Most AEs were grade 1–2 and could be improved by symptomatic treatment. Grade 3 AEs in this study mainly included hypertension (3/28), fatigue (2/28), anorexia (1/28) and vomiting (1/28), all of which improved after symptomatic treatment or drug dose adjustment. The overall incidence of severe AEs in this study was low, which is consistent with the results of other studies [11, 28, 29]. As expected, the most common side effect was hypertension, with a high incidence of 71.4%. This may be attributed to the multi-target antiangiogenic nature of anlotinib, which could elicit alterations in nitric oxide, endothelin-1, microvascular rarefaction, selective vasoconstrictions and renal injury, all of which have been cited as potential mechanisms leading to anlotinib-induced hypertension [28]. Neurocognitive impairment is also an area of concern during WBRT. In this single-arm study, the most prominent cognitive dysfunction observed was impairment of short-term memory. In this study, 13 out of 28 included patients maintained intracranial lesion control for more than 12 months. Among these patients, eight reported, either personally or through their family members, a decline in short-term memory, although this did not significantly interfere with their daily lives. The impact of anlotinib or other anti-angiogenic drugs on neurocognitive functions in patients undergoing WBRT warrants further investigation in larger cohorts.

Although the results of this study demonstrated the promising efficacy and safety of anlotinib in combination with WBRT for patients with multiple BMs from NSCLC, there are several limitations. First, the sample size was still small. The limited number of cases increased the statistical uncertainty of the survival time results. Second, the patients included in this study were all treated with anlotinib as a 3rd-line treatment, with no specific requirements for previous treatments or driver gene mutation status. The heterogeneity of the enrolled patients was high, making it difficult to analyse the effects on a specific patient subtype. Third, extracranial lesion regression was not monitored in detail at follow-up in this study, as this outcome was not the focus of this study and has been investigated in numerous clinical trials [11, 30].

In conclusion, our 2-year follow-up results show that anlotinib combined with WBRT is a promising combination therapy with good efficacy and tolerability in patients with NSCLC with multiple BMs. Our results can guide the design of future randomized controlled trials and clinical practice.

## Supplementary Material

Supplemental Material

## Data Availability

Research data are stored in an institutional repository and will be shared upon request to the corresponding author.
